# Trypanosomes, with Special Reference to Surra1Read before the McFarland Pathological Society of the Medico-Chirurgical College.

**Published:** 1903-02

**Authors:** Walter G. Bowers

**Affiliations:** Student, Medico-Chirurgical College, Philadelphia, Pa.


### THE JOURNAL

OF

### COMPARATIVE MEDICINE AND VETERINARY ARCHIVES.

VOL. XXIV. *FEBRUARY, 1903.* No. 2.

### TRYPANOSOMES, WITH SPECIAL REFERENCE TO SURRA.[1]

WALTER G. BOWERS, STUDENT,
MEDICO-CHIRURGICAL COLLEGE, PHILADELPHIA, PA.

THE subject of my paper is trypanosomes, with special reference to the disease called surra, which is epidemic in the Philippines at present, and, as a result, our government, with its faithful workers, are clearing up many of the dark problems concerning the etiology and pathology of this disease, which is of interest to us as scientific students of comparative medicine. The family trypanosomidæ, germs trypanosoma, of which there are many species, belongs to the flagellate class of protozoa. A broad term that has lately been introduced into comparative medicine to designate any infection of any animal with parasites belonging to the flagellate family trypanosomidæ is trypanosomiasis. This will no doubt lessen the confusion brought about by some sixty vernacular names which have appeared in the medical literature upon this subject, based principally upon prominent symptoms of the disease in question. There are several different kinds of trypanosomiasis, among which we may mention the following in particular: 1. Surra, a disease of horses, camels, elephants, and certain other animals of India, where it is endemic; it is attributed to trypanosoma Evansi. 2. Nagana, or tsetse fly disease of Africa, affecting cattle, horses, mules, asses, antelopes, camels, and domestic animals, and is attributed to trypanosoma Brucii. 3. Rat trypanosomiasis, attributed to trypanosoma Lewisi, which seems to be identical with the surra organism. No doubt the rat is capable of acting as an intermediate host for all species of trypanosoma. The life cycle of the different species of trypanosoma

[1] Read before the McFarland Pathological Society of the Medico-Chirurgical College.

(if there be such) has not been carefully studied as yet, but it is highly probable that the cycle of changes that the parasite undergoes simulates that of the malarial parasite of man in many respects, in that there is an asexual and sexual stage of development with symbiosis or sexual conjugation and resulting zygote stage in a definitive host, as one or more species of tabanidae (gad flies). The parasite of surra, or trypanosoma Evansi, resembles a whip-like worm. Its length is from 10 to 12 microns, and is from 1 to 1.5 microns in diameter through its body. The neck is nearly one-half its length, tapering gradually to a point representing the mouth. It has a limiting membrane, which is well-defined. The contour in most cases is symmetrical, but in some the body-line is quite irregular. The body, which is the larger part of the parasite, contains granular material and clear spaces, or vacuoles, which latter vary in size and number. The granular material does not extend to the neck. The parasite is actively motile, having both a vermicular and spiral movement. It moves forward in a very peculiar manner. The long, whip-like process is thrust forward by a spirillar motion, followed by a contraction of the body. Two sizes of the parasite have been observed. The larger appears to be more numerous and contains considerably more granular material than the smaller, and usually two or more vacuoles, which, no doubt, are pulsating and give rise to protoplasmic currents. Whether these two sizes represent male and female has not been determined; however, the union of the large and small parasites has been observed in such a way as to lead the observer to think that such union was something more than accidental. The organism appears to be a strict parasite, as it lives but a short time after removal from the body. The longest time which it has been kept in blood serum was not more than ten hours. It is easy to detect in microscopic examination of fresh blood films and readily takes the nuclear stains. The pathological change caused by this parasite is a rapid destruction of red corpuscles, causing an acute and profound anæmia. The changes occur in the blood coincident to the invasion of the parasite. In one horse that had been ill seven days the red blood cells numbered 3,500,000, the white cells 14,000. The blood count of a healthy horse taken as comparison gave red blood cells 6,900,000, white cells 9800. There is also a slight oligochromæmia, however, as in pernicious anæmia. There is a greater amount of hæmoglobin in each red blood cell than during health. Surra has been known in India for several generations. The attention of the rest of the world was called to this disease in a report to the government of India by Dr. Griffith Evans in the fall of 1880. Since then numerous papers on the subject have been written by English veterinarians and physicians, and it was not until the present epidemic in the Philippines that our government has taken up the study of trypanosomatic diseases. It is not positively known whether surra has existed in the Philippines many years or not. If of recent introduction, one of the explanations is that it was carried to China by the English troops from India, and that our troops carried it from China to the Philippines. However, this explanation is not in accord with the statements made by Drs. Slee and Kinyoun, who claim that surra was present in the Philippines before our troops went to China. It has long been observed that surra is a disease that is prone to appear and spread during or immediately following heavy rainfalls; also, that it is more common in the lowlands in the vicinity of water, as canals and rivers; however, sporadic cases may occur in the dry and hot seasons of the year. From these facts flies were given some study. In dry weather biting flies are not so active as in moist weather, and flies which are in a pupal stage are not so likely to issue from their dormant condition. However, with the advent of such conditions that prevail during the rainy season, flies which are in a pupal stage come forth from the dry ground and proceed to breed rapidly. Thus during and following rainfall there would be more biting insects flying; hence there is a greater chance of spreading the disease by infection.

With reference to sex this plays no rôle, as horses and mares are affected with surra alike. Australian horses are much more subject to the disease than are the Asiatic horses, and it is said that white and gray mules are more susceptible than darker animals; at least, flies attack light-colored animals more than they do dark, as the mosquito attacks whites more than they do negroes. Surra is not confined to horses, asses, and mules alone, but outbreaks of the disease are reported also among camels, elephants, cats, and dogs, and the disease has been transmitted by inoculation to cattle, buffalos, sheep, goats, rabbits, guinea-pigs, rats, and monkeys. The limits of its infectivity are not yet established, and thus for the present all mammals must be considered as possible carriers of the disease until negative experiments prove them to be refractory. No birds or amphibians are known to harbor the parasite. Nearly all authors agree that surra in horses and camels is invariably fatal, and although some few cases are said to have made slow progress in the disease, alternating better and worse for over a year, they all eventually succumb. Burke says that there is probably no disease in which the mortality is so high as in pernicious anæmia, which he identifies with surra. One author has seen all the dogs of a village swept off by this disease after eating surra carcasses. Cats also take the disease in the same way. Whether the dogs were actually infected through eating the meat, or whether the carcasses had attracted flies which in turn had bitten the dogs, seems to be an open question. However, both explanations seem plausible, for the parasites might enter the dogs through some wound in the mouth such as could easily be made while gnawing bones, as experiments carried out by Rodgers show that if surra blood is fed to an animal with an abraded mucous membrane with which the blood comes in contact, infection will take place; *per contra*, if there is no abrasion no infection follows. However, it seems possible that the parasites might force their way through the intestinal mucosa, as the trichinæ spiralis and tubercle bacillus.

Opinions differ as to the source of infection by the parasite of surra. While the drinking water and grain-containing feces of rats have been viewed with suspicion by prominent authorities, most authors now believe that flies of the family tabanidæ (gad flies) act as transmitters of the organism. This latter view is strongly supported by analogy, for nagana, which is also caused by a trypanosome—and some writers consider nagana as nearly, if not quite, identical with surra—is carried by the tsetse fly, and trypanosomiasis of rats have been shown to be transmitted by fleas, while lice may also disseminate the same parasite. Lingard demonstrated that the body fluids of certain flies, after sucking the blood of surra animals, contained trypanosoma in varying numbers; and, further, that healthy animals, when inoculated with such material, contracted the disease after a period of incubation.

These facts, taken in connection with the recent experiments on nagana and rat trypanosomiasis, indicate very strongly that surra is transmitted by dipterous insects, as gad flies. The fly theory of surra explains satisfactorily a number of points which have long been observed in connection with this disease, that is, that surra is a disease of wet weather, as has already been pointed out, and biting flies are practically creatures of moisture and breed very rapidly in wet weather. Howard, in discussing the fly family, some of which are the transmitters of surra, if not the definitive host for trypanosoma Evansi, says: "The adult flies are great water drinkers, and are usually most abundant in the vicinity of inland ponds and streams."

Noting this fact, Porchinsky, the Russian entomologist, has destroyed great numbers of the larvæ of flies by coating such ponds with kerosene. The larvæ live in the earth or in water, and are carnivorous, feeding upon soft-bodied insects and water snails. The spindle-shaped brown or black eggs are deposited in summer in groups attached to the leaves or stems of herbage. The gad flies vary greatly in color and size, of which there are ahout 1500 species, 200 of which occur in this country, and the largest of which is tabanus Americanus, which is 1¼ inches long and has a wing-spread of 2½ inches.

The fly that seems to be the common transmitter of surra in the Philippines is stomoxys calcitrous, leg-sticker, or common biting stable-fly, in which has been repeatedly found the parasite of surra, both in the stomach and proboscis. The few experiments that have been carried out thus far as to a life cycle of the parasite in this fly have been negative, and if there is such it is short. Birds have been shown to be transmitters of surra by their blood-stained beaks and soiled feet, with undried trypanosomatic blood coming in contact with a raw surface, as galls of other animals. This shows, as in malaria, that the blood containing the parasites can be directly conveyed to another animal and cause the disease. Congenital infection is not known. Infection from drinking stagnant water and eating vegetation grown upon land subject to inundation seems to be of little moment as an etiological factor in the production of surra, as Pease has observed the most careful precautions taken as to boiling the water and feeding nothing but select hay and grain to the animals—in fact, all imaginable precautions were taken to prevent the introduction of the disease—and still the disease appeared and every single animal perished.

It seems that the most probable transmitter of the disease was lost sight of, as they were not thoroughly protected from the suctional horsefly. As to the clinical symptoms of surra, there is no history of a definite onset. There is rarely an opportunity to note early symptoms, as the attack is a mild one and we may not notice such prodromes as that of the horse. Being somewhat dull and listless, he may be off his feed for a few days and stumble during action, and may be leg-weary, but after a few days' rest the animal apparently recovers. The period of incubation is apparently subject to considerable variation, and may be from six, ten, to twenty days. With the true invasion the prodromal symptoms are exaggerated and the skin is hot and dry, and we have anorexia and polydipsia as prominent symptoms. Temperature more or less elevated; pulse full and frequent, 56 to 64 beats per minute. The mucosa of the conjunctiva, especially that covering the membrana nictitans, is the seat of red patches of ecchymoses. There are catarrhal symptoms, with excessive lacrymation and mucous discharge from the nostrils (coryza), with snuffling breathing. There may be a general or local urticarial eruption. There is more or less œdema of the limbs about the fetlocks and hocks and beneath the belly and chest. This dropsical swelling sometimes subsides for a time. There is some glandular enlargement, especially the submaxillary glands. There may be ulceration of the nasal mucous membrane, tongue, and buccal cavity. The genitaliæ are markedly affected in this disease. In mares the mucous membrane of the vagina is decidedly yellow, and there are numerous petechia on the vulva and in the vagina, and frequent symptoms of horsing, due, no doubt, to the mechanical irritation that is kept up in these parts.

The parallel to this sexual phenomenon of mares is seen in male animals, who are visited with frequent and uncalled-for attacks of priapism, which may be due to irritation of the genito-spinal centre in the cord, but no doubt is associated with thrombi in the erectile tissue of the penis, or there may be embolic plugging of the vessels. Nervous symptoms are present, as stringhalt, dragging of the hind limbs, muscular tremors, and in some cases paralysis of the hind quarters. Gurgling and tympanitis are prominent symptoms. Respiration is usually accelerated from the first and breathing appears to be entirely abdominal. Cheyne-Stokes respiration has been noted toward the last in a few cases. The pulse is accelerated at first. It ranges between 40 and 90 beats per minute, and may reach as high as 120 just before death. It tends to be frequent during the paroxysms of fever, but during the apyrexial period falls to about normal. The fever is of an intermittent and remittent type, and somewhat resembles the fever of malaria in its behavior. However, the paroxysms of fever are much longer in surra and appears at irregular periods of time. The duration of surra varies with the species of animal affected, their age and general condition, the average duration being about two months, when death occurs from general asthenia. Immediate death is often brought about by ante-mortem clot in the heart, or simply failure of the heart's action as a result of debility. Post-mortem examination does not reveal any organic changes in the liver, kidneys, heart, spleen, lungs, and mucous membrane of stomach and intestines. No doubt most of the changes are of a functional character, due to the profound anæmia. Rigor mortis is normal, and the animal presents a dreadfully emaciated appearance. The mucous membranes are of a yellow color, while the muscles are dark red in color; in short, most of the changes simulate those of pernicious anæmia. Pneumonia, nephritis, pleurisy, and pericarditis often enter in as complications, and tend to hasten the end.

As to the treatment of surra, it is prophylactic rather than curative, as our most powerful parasiticides and drugs that are destructive to lower forms of life have been used with negative results. However, arsenic in the form of liquor potassi arsenitis given intravenously has been found to be destructive to the parasites in a few cases. No doubt its effect as a hæmaturic and glandular stimulant increasing the number of red corpuscles and the amount of hæmaglobin, thereby lessening the progressive and destructive anæmia and its tonic impression on the nerve centres and mucous membranes, overshadows its destructive action on the parasites, which is evanescent in character. The treatment which demands our attention, as in all other diseases of this nature, is prophylaxis, and in closing depends upon, first, isolation and quarantine of infected animals or animals suspected of infection, and protecting them from flies, if they have wounds or sores of any kind; second, protection of stocks from flies in wet weather by smearing and by the use of smudges; third, and most important, is to exterminate the transmitter by preventing flies from breeding by doing away with the outside manure-pile and by the free use of kerosene and chlorinated lime.

